# Effect of Photobiomodulation Therapy on Oxidative Stress Markers in Healing Dynamics of Diabetic Neuropathic Wounds in Wistar Rats

**DOI:** 10.1007/s12013-021-01021-9

**Published:** 2021-07-30

**Authors:** Gagana Karkada, G. Arun Maiya, Praveen Arany, Mohandas Rao, Shalini Adiga, Shobha Ullas Kamath

**Affiliations:** 1 Scholar, Centre for Diabetic Foot Care and Research (CDFCR), Department of Physiotherapy, Manipal College of Health Professions, Manipal Academy of Higher Education, Manipal, Karnataka 576104 India; 2 Chief-Centre for Diabetic Foot Care and Research (CDFCR), Professor-Department of Physiotherapy, Dean-Manipal College of Health Professions (MCHP), Manipal Academy of Higher Education, Manipal, Karnataka 576104 India; 3 Department of Oral Biology School of Dental Medicine, Engineering & Applied Sciences, University at Buffalo., 3435 Main Street, B36A, Foster Hall, Buffalo, NY 14214-8031 USA; 4 Head of the Department-Department of Anatomy, Melaka Manipal Medical College-Manipal Campus, Manipal Academy of Higher Education, Manipal, Karnataka 576104 India; 5 Head of the Department, Department of Pharmacology, Kasturba Medical College, Manipal Academy of Higher Education, Manipal, Karnataka 576104 India; 6 Department of Biochemistry, Kasturba Medical College, Manipal Academy of Higher Education, Manipal, Karnataka 576104 India

**Keywords:** Diabetic wounds, Catalase, Malondialdehyde, Oxidative stress, Photobiomodulation therapy, Wound healing

## Abstract

**Background:**

Prolonged and overlapping phases of wound healing in diabetes are mainly due to the redox imbalance resulting in the chronicity of the wound. Photobiomodulation therapy works on the principle of absorption of photon energy and its transduction into a biological response in the living tissue. It alleviates the cellular responses, thereby improving the mechanism of wound healing in diabetes.

**Objective:**

To find out the effect of photobiomodulation therapy of dosage 4 J/cm^2^ in the healing dynamics of diabetic neuropathic wounds in Wistar rats and its relation with oxidative stress markers.

**Methodology:**

Diabetes was induced using Streptozotocin of 60 mg/kg of body weight to eighteen female Wistar rats. Neuropathy was induced by the sciatic nerve crush injury followed by an excisional wound of 2 cm^2^ on the back of the animal. Experimental group animals were treated with dosage 4 J/cm^2^ of wavelength 655 and 808 nm, and control group animals were kept unirradiated. The biomechanical, histopathological, and biochemical changes were analysed in both groups.

**Results:**

There was a reduction in mean wound healing time and an increased rate of wound contraction in the experimental group animals compared to its control group. The experimental group showed improved redox status, and histopathological findings revealed better proliferative cells, keratinisation, and epithelialization than un-irradiated controls.

**Conclusions:**

Photobiomodulation therapy of dosage 4 J/cm^2^ enhanced the overall wound healing dynamics of the diabetes-induced neuropathic wound and optimised the oxidative status of the wound, thereby facilitating a faster healing process.

## Introduction

Diabetes mellitus (DM) is a leading non-communicable disease in India and Globally. Individuals with DM suffer from microvascular and macrovascular complications that predispose them to develop conditions like cardiovascular diseases, peripheral vascular diseases, retinopathy, nephropathy, and peripheral neuropathy [1]. Diabetic foot ulcer (DFU) is the most complex yet neglected complication of diabetic peripheral neuropathy (DPN). The intrinsic and extrinsic factors of DPN makes the feet vulnerable to injuries and tissue damage, causing chronic wound or ulcer [2]. The global burden of DM affected nearly 463 million people in 2019 and is anticipated to rise up to 700 million by the end of 2045. During the 2019 survey, India reported the prevalence of 72 million people affected by DM and an anticipated increase of up to 153 million by 2045. Globally, foot ulcers occur in 15–25% of people with DM and between 5–7.5% of those with neuropathy [3].

Delayed wound healing in DM is a major secondary complication that often ends with the loss of limb and disability. Regular wound repair progresses through the discrete phases of inflammatory, proliferative, and remodelling. In DM, these phases are prolonged and overlapped due to underlying excessive tissue damage, tissue perfusion, and lack of oxygenation that delay the wound healing process [4]. The tissue renewal process depends on the level of oxygen (O_2_) at the wound bed. The fundamental cellular process where oxygen is involved in the oxidative phosphorylation in mitochondria, mainly yields adenosine triphosphates (ATPs). The derivatives of O_2,_ known as reactive oxygen species (ROS) produced during mitochondrial ATP production, act as signalling molecules in tissue repair. The low-level ROS results in cell cycle arrest, increased levels result in oxidative damage and cell apoptosis. Therefore, the optimum levels of ROS molecules are required for the normal tissue repair process [5]. The homoeostasis of ROS molecules generated during wound healing is by the special group of molecules known as antioxidants. These antioxidants have the capacity to significantly delay or prevent oxidative action of ROS and mitigate its deleterious effects. The well-known antioxidants are glutathione, superoxide dismutase, and catalase etc. [6].

Catalases are the haem containing antioxidant enzyme mainly produced in nuclear peroxisomes and mitochondria that catalytically decompose Hydrogen peroxide (H_2_O_2_) into oxygen and water using iron and manganese as co-factors. It can reduce the concentration of H_2_O_2_ at a rapid rate without consuming cellular energy, thereby providing cell defence against oxidative damage. However, in diabetic wound healing, catalase activity is decreased, leading to increased H_2_O_2_ concentrations in the blood and tissues [7, 8]. The failure of redox balance at the wound site results in delayed cellular responses, failing the wound recovery.

Photobiomodulation therapy (PBMT) is a promising conservative non-pharmacological approach in the management of diabetic wound healing. PBMT works on the principle of low energy biostimulation, causing photochemical reactions in the cells/tissue [9]. Studies have explored the effectiveness of PBMT in diabetic wound healing in vitro, in vivo, where PBMT of dosage 2–6 J/cm^2^ had shown a beneficial effect in wound healing dynamics [10–12]. PBMT is believed to alter enzyme activation and cell cycle progression, thereby affecting cells’ redox-sensitive transcription factors. PBMT enhances antioxidants production by enzymatic reactions, which are the fundamental mechanisms involved in wound healing [13].

Although there are studies involving the mechanism of PBMT in wound healing, very few studies have elucidated the dose-dependent mechanism of PBMT and their relation with oxidative markers in the healing dynamics of diabetic neuropathic wounds. Therefore the present study aims to find out the effect of PBMT of dosage 4 J/cm^2^ in the healing dynamics of diabetes-induced neuropathic wounds in Wistar rats, with biomechanical, histopathological, and biochemical finding which may add substantial evidence to the existing literature of wound healing in diabetes.

## Material and Methods

### Animal Selection and Care

We obtained the institutional animal ethics committee approval before the study’s initiation (IAEC/KMC/95/2018). In house bread, eighteen female Albino Wistar strain rats of weight (220.78 ± 11.87) g and age (5.43 ± 0.11) months were procured from the institutional central animal research facility. The animals were housed in propylene cages (29 × 22 × 14 cm) with paddy husk bedding. Their living environment was maintained with temperature 28 ± 1 °C, humidity 55 ± 5%, and 12 h of light and dark cycle. The animals were provided with sufficient food once in the day and water *ad libitum*.

### Induction of Diabetes

Baseline blood glucose levels were measured using calibrated Accu-check Performa Nano glucometer (Roche diagnostics India). Diabetes was induced to all the animals by intraperitoneal injection of Streptozotocin (STZ) (MP Biomedical^TM^ India) of dosage 60 mg/kg body weight dissolved in 0.1 M citrate buffer of pH 4.5 (1 mL) [14]. Food and water were provided after 30 min of injection. Further, the animals were placed in individual metabolic cages to observe clinical and behavioural changes of DM for seven days.

### Confirmation of Diabetes

The animals were observed for polydipsia, polyphagia, and polyuria, and changes were recorded. Besides, the weight of the animals was recorded on alternative days. On the 7th day, blood was drawn in the fasting state from the intra-orbital plexus by inserting a capillary. Blood (1.5 mL) was drawn into the test tube containing sodium fluoride. The sample was mixed thoroughly and allowed to clot for 20 min. The sample was then centrifuged at 2000 × *g* for 10 min, and the supernatant serum was collected. Blood glucose levels were estimated using the glucose-oxidase and peroxidase method (GOD-POD kit, Coral clinical systems India). Animals with fasting blood glucose levels of ≥200 mg/dL were included in the study. Blood glucose levels were monitored periodically using calibrated Accu-check Performa Nano glucometer.

### Induction of Neuropathy by Sciatic Nerve Injury

We followed previously established protocols developed for the neuropathic model in all the animals [15, 16]. Under expert supervision, the animals were anaesthetised with intravenous Ketamine of dosage 2 mg/kg body weight. The right/left hind leg from the knee to the hip was shaved using an electric shaver. An incision was made ~0.5 cm parallel to the femur and about 1.5 mm anterior to the femur. Under sterile conditions, the sciatic nerve was revealed until mid-thigh level and crushed for 20 s with the tip of watchman forceps (2 × 15 s) to induce neuropathy.

### Confirmation of Neuropathy

The animals were kept for three weeks of the stabilisation period. Their clinical and behavioural changes for neuropathy were recorded. The walking, scratching, grooming, resting/sleeping, eating, and freezing activities were observed. The following tests were performed to confirm the neuropathy in the feet.

#### 5.07 monofilament (10g) test

Semmes Weinstein 10 gm monofilament is used in human participants for the detection of loss of protective sensation. A slightly modified procedure was performed on the animal’s feet [17]. Here, the animals were placed on a metal mesh grid. Monofilament was applied perpendicularly with a force of 10 g from the lower surface of the mesh to the plantar areas of the hind paws. The time taken in seconds by the animals to withdraw paw in response to monofilament touch was noted. In the normal feet, The time taken for the paw withdrawal was between 1–4 s, whereas the neuropathy induced feet showed a delayed response.

#### Hind paw withdrawal test for hot stimulus

We followed the previously established procedure for hind paw withdrawal to confirm the neuropathy [16]. The animals were placed on a metal hot plate (45 ± 0.5° C) of dimensions 15 × 15 cm for an evaluation period of 3 min. The total duration for responding to the hot stimulus by each animal to lift neuropathy induced and non-induced leg was noted. In the non-induced feet, The time taken for the paw withdrawal was between 1 and 4 s, whereas the neuropathy induced feet showed a delayed response.

#### Hind paw withdrawal test for cold stimulus using acetone

The slightly modified procedure from the previous study was conducted [18]. The animal was placed upon a metal mesh grid with open access to the paws from below. A cotton swab was dipped in acetone and was brought into the plantar surface of the hind feet. The time taken to withdraw the leg from the supporting mesh after the exposure to acetone was noted. Both normal and neuropathy-induced legs were exposed to acetone, and the test was performed for 3 min. The response within 4 s was considered a normal, brisk response to differentiate from the altered lifting.

On the final day of PBMT intervention, the tests were repeated to evaluate to determine the changes in their neuropathic state.

### Excisional Wound Model

An excisional wound model was created on the back of the animals near the neuropathy induced leg. Animals were anaesthetised using intravenous Ketamine of 2 mg/kg body weight. The wound area to be created was marked using a circular rubber stamp of area 2 cm^2^ dipped in methylene blue. The dorsal fur was shaved, and an excisional wound was created along the markings using toothed forceps using number 21 surgical blade and pointed scissors [11]. The area of the wound was recorded on transparent sheets. After the procedure, each animal was kept in a separate cage, and all wounds were exposed to air.

### Animals Grouping

The animals were assigned into two groups, i.e. the control and experimental groups, based on the animals’ weight and blood glucose levels.

I. Control group *N* = 9 (diabetic neuropathic wounded-unirradiated)
II. Experimental group *N* = 9 (diabetic neuropathic wounded-irradiated with 4 J/cm^2^)

### Photobiomodulation Therapy

After calibration of the equipment Tech laser SS 100 (Technomed Electronics Chennai), the standard protocol for the laser irradiation was followed [11]. The experimental group animals were treated with PBMT of dosage 4 J/cm^2^. A Combination of scanning and probe laser was given once a day to the experimental group animals until the complete closure of the wound (~2 weeks). The control group animals were kept un-irradiated. Specifications of PBMT used in the study are described in Table 1.

**Table 1:** Characteristics of PBMT used in the study

PBM	Wavelength (nm)	Wave emission	Power output (mW)	Power Density mW/cm^2^	Spot size (cm^2^)	Dosage J/cm^2^	Depth cm	Duration min
Scanning laser	655 Visible red	Continuous	24	2.64	9.1	4	1	6
Probe laser	808 Infra-red	Continuous	120	120	1	4	3	3

### Biomechanical Analysis of Wound

#### Wound area measurement

The wound areas were recorded on a transparent sheet. The area was measured using ImageJ software (Downloaded from https://imagej.nih.gov/ij/download.html Windows Download ImageJ bundled with 64-bit Java 1.8.0_172). The total area was represented in cm^2^.

Mean wound healing time was calculated by taking the sum of the number of days taken for complete wound closure by each animal in the control and experimental group. Data was reported as Mean ± SD (*N* = 9 in each group). Serial photographs were taken for observation of wound closure on day-1, 7 and 16.

#### Rate of contraction of wound

The rate of contraction of the wound was calculated using the formula [initial area (I)-final area (F)/number of days (N)] per day cm^2^ [11].

#### Histopathological analysis

The tissues were processed on day 16th from both the experimental and the control group and

fixed in formalin (10%) for further histopathological analysis. As per standard laboratory protocol, the tissue samples were dehydrated with ascending grades of alcohol. The samples were then transferred to Xylene to clear the remaining alcohol. The tissue was embedded and moulded in paraffin wax. The 5-μm thick sections were obtained using a microtome, and clean sections were fixed to slides. The slides were stained with H&E staining to observe epithelialization and distribution of collagen fibres [19]. Each section was then viewed under the light microscope (Lx 300, Labomed, USA).

#### Immunohistochemical analysis

Immunohistochemistry was performed for the qualitative analysis of proliferative cells using Anti-Ki-67 antigen, Proliferating Cell [BioGenex, California], to compare the cell proliferation in the control and the experimental group. The previously established protocol was followed for immunohistochemical staining for Ki-67 antigen-specific proliferative cells [20]. Each section was then viewed under the light microscope (Lx 300, Labomed, USA).

### Biochemical Analysis

#### Estimation of antioxidant catalase assay

Catalase activity was determined according to Beers and Sizer by spectrophotometric monitoring of hydrogen peroxide decomposition by hemolysates at 240 nm at room temperature [21]. Results were expressed in units/g of haemoglobin

#### Lipid peroxidation by malondialdehyde (MDA) assay

MDA is a highly reactive three-carbon di-aldehyde produced as a by-product of polyunsaturated fatty acid peroxidation and arachidonic acid metabolism. Its level indicates the oxidative status of the cells. Serum MDA was measured using Kei Satoh’s Method of spectrophotometric determination [22]. Results were expressed in nmol/g haemoglobin.

### Statistical Analysis

Descriptive statistics were measured using EZR (R version 3.4.1 (C) 2017 The R Foundation for Statistical Computing), and data were expressed in (Mean ± SD). For the normally distributed data, Paired sample *t*-test was used. When the data were not normally distributed, the non-parametric Wilcoxon signed-rank test was used. *P* < 0.05 was considered statistically significant.

## Results

### Metabolic Cage Analysis of Diabetes-induced Animals

**Fig. 1:**
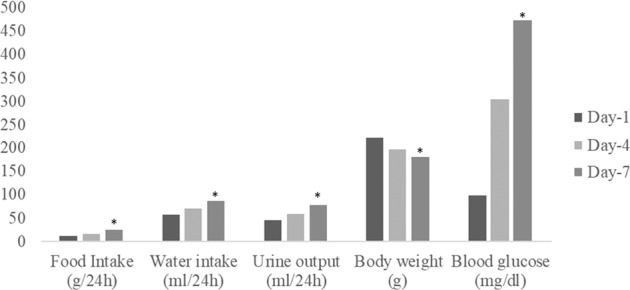
Demographics and details of metabolic cage analysis of STZ induced animals (*N* = 18) (*p* value < 0.05 significant*). 1. Food intake (g) 2. Water intake (mL) 3. Urine output (mL) 4. Body weight (g) 5. blood glucose (mg/dl).

Our study findings revealed that, after the induction of diabetes using STZ, all the animals showed a significant decrease (*p* = 0.015^−6^) in their bodyweight. The weight reduction was gradual from day-1 of the induction of diabetes until day-7. There was a significant increase in the blood glucose (*p* = 0.015^−6^), water intake (*p* = 0.081^−6^), Urine Output (*p* = 0.039^−6^), and food intake (*p* = 0.039^−6^) by the end of the day-7. These symptoms are the classical triads of diabetes mellitus. The animals also displayed irritability, gradual hair loss, and skin colour changes upon diabetes induction. On day-7, the blood glucose levels of the animals were >200 mg/dl (Fig. 1).

### Confirmation of Neuropathy

There was a significant change in the paw withdrawal time in the neuropathy-induced leg compared with the non-induced leg. The recorded responses to the Monofilament test, hot stimulus, and cold stimulus in the neuropathy-induced feet are listed in Table 2. The comparison between neuropathy induced leg to that of the non-induced leg confirms the sciatic nerve’s damage and affected microcirculation. The observational findings also revealed a change in their resting, walking, and scratching pattern in the neuropathy-induced leg.

**Table 2:** Response to neuropathy confirmation tests (*N* = 18)

Confirmatory tests	Time-taken for response in sec Neuropathy induced feet	Time-taken for response in sec Non-induced feet
1. 10 g Monofilament	7.11 ± 1.02	3.66 ± 0.59*
2. Hind paw withdrawal test for hot stimulus (45 ± 0.5 °C)	6.6 ± 0.76	3.05 ± 0.53**
3. Hind paw withdrawal test for cold stimulus using acetone	6.22 ± 0.7	2.5 ± 0.51***

Values expressed in (Mean ± SD)

*,**,*** represents the significant *p* values 0.17^−3^, 0.18^−3^, 0.15^−3^ respectively

### Biomechanical Analysis

#### Mean wound healing time

**Fig. 2:**
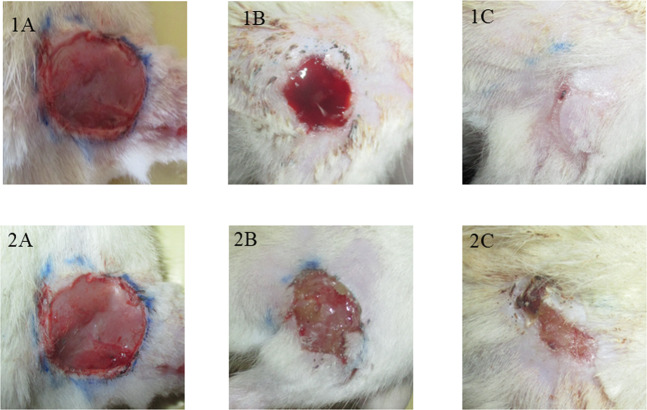
Representative image of wound healing in experimental and control group animals. 1) Representative photographs of wound healing in experimental group in pictures 1**A** (Day-1), 1**B** (Day-7) and 1**C** (Day-16) group. 2) Representative photographs of wound healing in control group in pictures 2**A** (Day-1), 2**B** (Day-7) and 2**C** (Day-16) group

The wounds were photographed to observe the healing nature on day 1, 7 and the final day of healing. The experimental group showed complete wound healing with a mean of (16.12 ± 0.64) days. The unirradiated control group showed a mean of (21.62 ± 0.51) days for complete healing (Fig. 2).

#### Wound contraction

Our study’s findings revealed that the wound closure was better and faster in the experimental group treated with 4 J/cm^2^ than the un-irradiated control groups. There was a significant decrease (*p* = 0.013) in mean wound healing time in the experimental group compared to the control group. The rate of wound contraction was also significantly higher in groups treated with 4 J/cm^2^ (*p* = 0.014) when compared to its un-irradiated controls (Table 3).

**Table 3:** Rate of wound contraction in the animals (*N* = 9 in each group).

Groups	Initial area (cm^2^) Day-1	Final area (cm^2^) Day-16	Number of days for healing (N)	Rate of wound contraction/Day (cm^2^)
Control group	2.0 ± 0	0.45 ± 0.03	21.62 ± 0.51	0.07 ± 0.04
Experimental group (4 J/cm^2^)	2.0 ± 0	0.13 ± 0.008	16.12 ± 0.64*	0.11 ± 0.13**

Values expressed in (Mean ± SD).

*, ** represents the significant *p* values 0.013, 0.014, respectively

#### Post PBMT neuropathy assessment

The neuropathy tests performed on day 16th revealed that the experimental group animals treated with PBMT of dosage 4 J/cm^2^ showed significant change in paw withdrawal time in the neuropathy induced feet. The response to monofilament, hot stimulus, and cold stimulus was significantly improved in neuropathy-induced feet compared to its un-irradiated control group (Table 4).

**Table 4:** Post neuropathy assessments (*N* = 9 in each group).

Tests	Control group	Experimental group (4 J/cm^2^)
	Time taken response in sec neuropathy induced feet	Response in sec in neuropathy induced feet
1. 10 g Monofilament	7.44 ± 0.72	6.66 ± 0.70*
2. Hind paw withdrawal test for hot stimulus (45 ± 0.5 °C)	6.88 ± 0.78	6.33 ± 0.86**
3. Hind paw test for cold stimulus using acetone	7.00 ± 0.70	5.55 ± 0.52***

Values expressed in (Mean ± SD)

*,**,*** represents the significant *p* values 0.80, 0.12, 0.012, respectively

#### Histopathological analysis

**Fig. 3:**
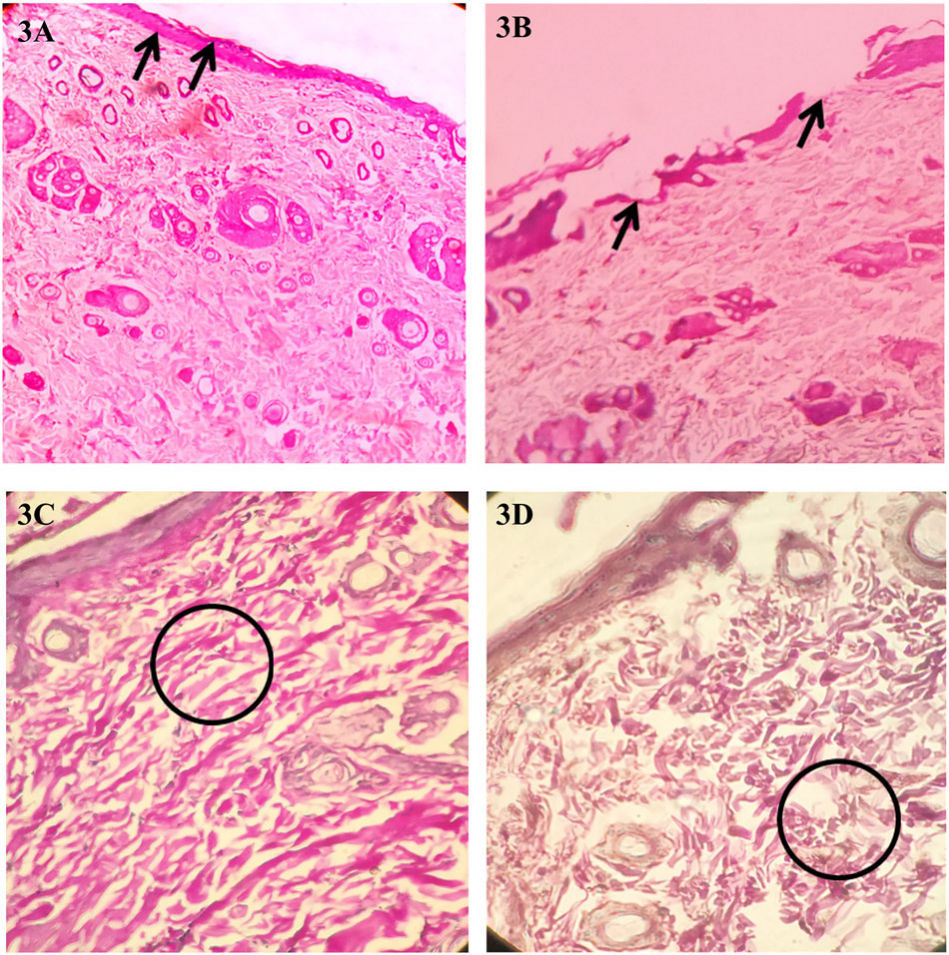
Representative photomicrographs of H&E staining of skin tissue of the experimental and control group. (1) Representative photomicrographs of H&E stained tissues from the wound area of experimental group treated with 4 J/cm^2^ (3**A**) showing better epithelialization than non-irradiated control group (3**B**) (marked with black arrows) under 10× magnification. (2) Experimental group (3**C**) and control group (3**D**) show the collagen fibres in the dermis. 3**C** represents more organizely arranged collagen fibres when copared to 3**D** (marked with black circle) under 40× maginification

The tissue samples were collected from the experimental group and the control group animals on day-16. The qualitative analysis of histopathology for H&E stained skin tissues from the wound area revealed a close resemblance to the normal skin. The Experimental group treated with 4 J/cm^2^ showed marked epithelialization and keratinisation, indicating a faster healing process when compared to the un-irradiated control group. (Fig. 3A, B). The density and structural organisation of collagen fibres in the dermal region showed better positioning in the experimental group when compared to that of the control group (Fig. 3C, D).

#### Immunohistochemistry

**Fig. 4:**
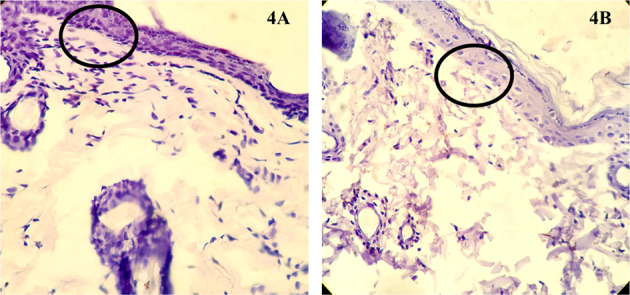
Representative photomicrographs of Immunohistochemical analysis for Ki-67 antibodies for proliferative cells. Representative photomicrographs of immunohistochemical analysis of the tissues from the wound stained for IHC-Ki67 antibody for proliferative cells. 4**A** Experimental group treated with 4 J/cm^2^; 4**B** Control group. It can be noted that the experimental group showed well-stained nuclei indicating well proliferative cells especially near the stratum basale of epidermis when compared to control group (marked with black circle)

The experimental group and control group’s immunohistochemical analysis revealed that the experimental group treated with 4 J/cm^2^ (Fig. 4A) showed well-stained nuclei, especially in the basal layer (stratum basale) of the epidermis indicating high proliferative activity in these cells. Besides, the abundance of active cells (fibroblasts) looked relatively high in the skin’s dermal region in revealing the active production of collagen fibres compared to that of the un-irradiated control group (Fig. 4B).

#### Catalase and MDA levels

**Fig. 5:**
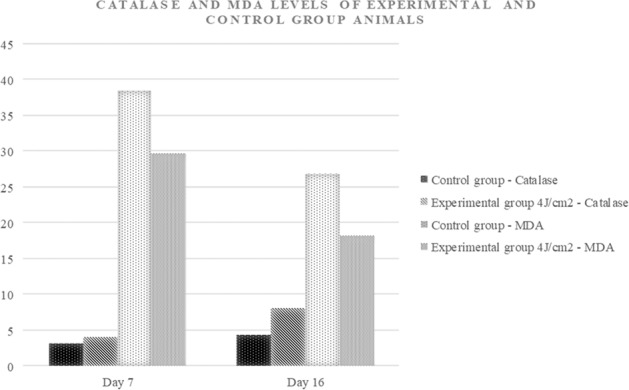
Catalase and MDA levels in the experimental and control group (*N* = 9 in each group).

In the present study, we observed a significant change in the catalase and MDA levels among the group and between experimental and control group animals on day-7 and day-16 (*p* = 0.004). A decreased catalase activity was observed in the un-irradiated control group compared with the experimental group treated with 4 J/cm^2^. On the contrary, we observed an increased MDA activity in control group animals compared to that of the experimental group on both day-7 and day-16 (Fig. 5).

## Discussion

DM is characterised by persistent hyperglycaemia that builds up the blood vessels’ sugar complexes, resulting in poor circulation and deranged nerve functions [23]. In the present study, the STZ-induced animals showed high blood glucose levels from day seven. The sciatic nerve injury to the animals demonstrated the degenerated nerve functions. The control and experimental group animals showed the typical polydipsia, polyphagia, polyuria, and gradual weight loss symptoms of DM. Akbarzadeh et al. demonstrated these characteristics of STZ induced animals [24]. The induced STZ destructs the islets of Langerhans beta cells, and as a result, clinical diabetes emerges in the animals.

Previous studies have described the different types of mechanical peripheral nerve injury and evaluations of neuropathic pain in human and animal models using the stimuli that give the degree of mechanical, chemical, and temperature-induced changes [17, 25, 26]. In the present study, we evaluated the neuropathic changes by 5.07 Monofilament (10 g) test, Hind paw withdrawal test for hot and cold stimulus where the neuropathy induced leg showed the delayed response. In Dowdall et al. study, the partial sciatic nerve ligation model produced a similar and significant change in lifting behaviour on the hot plate from minimal to the absence of lifting in the neuropathy-induced leg; however, the response depends on the type of sciatic injury [18].

There is evidence for the therapeutic role of PBMT in pain management, muscle repair, and nerve damage [27–29]. We also observed moderate improvements in the neuropathy-induced leg in the post-PBMT treated group compared with its control group, but the recovery was not complete. Bertolini et al. treated the animals’ sciatic nerve with low-level laser therapy (LLLT) of dosage 4, 6 J/cm^2,^ and 830 nm wavelength and found a significant change in paw-withdrawal time on the fifth day of therapy [30]. Anju et al. suggest that low-level laser therapy of dosage 3.1 J/cm^2^ significantly improved in painful diabetic neuropathy conditions [17]. PBMT can enhance the microcirculation of nerves, thereby improving the blood supply.

In the present study, the diabetic neuropathic wound models were treated with 4 J/cm^2^ of wavelength 655 and 808 nm for 6 and 3 min. The experimental group and the control group animals were kept unirradiated. We found that PBMT effectively accelerated the mean wound healing time and rate of contraction of wounds. Therefore, this significant improvement compared to the un-irradiated control group appears to be the direct effect of PBMT. Studies demonstrate the effect of PBMT of different dosages and wavelengths in chronic wound conditions like diabetic foot ulcers [31, 32]. Maiya et al. found that LLLT of dosage 3–6 J/cm^2^ and wavelength 655 nm accelerated the wound healing with significant improvement in wound contraction rate, whereas 7–9 J/cm^2^ decelerated the wound healing [11]. Lau et al. treated the diabetic excisional wound with LLLT of dosage 5 J/cm^2^ and 808 nm but of different power densities; found that PBMT significantly enhanced the percentage of wound closure [33]. The possible mechanism could be that, the photon energy delivered to the wound tissue is absorbed and produces biostimulation, thereby enhancing cell proliferation. Cell proliferation is characterised by neovascularization, epithelialization, and granulation tissue formation in post-inflammatory phases [9, 11].

We have analysed the histopathological changes in the experimental and control groups to justify these results. The experimental group’s H&E stained skin tissues showed better epithelium, collagen formation, and distribution than the control group. The immunohistochemical findings revealed that the proliferative cells were present abundantly in the experimental group than the control group, representing better and faster-wound healing. Therefore, it is evident that PBMT enhanced the formation of epithelial cells, fibroblastic cells, and proliferative cells, thereby enhancing epithelial tissue, granulation tissue, and collagen. Similar findings were also observed in Kilík et al. and Lau et al., who treated the diabetic mouse skin with PBMT [34, 35]. However, the present study did not obtain the skin at different stages of wounding. The mechanism of PBMT in the acceleration of wound healing could be by the mild inflammatory reaction induced by the PBMT, which enhances or upregulates neo-angiogenesis and increases the blood flow around the wound. On the other hand, the capacity of the PBMT to prevent harmful reactions during the inflammatory phase, facilitating collagen formation, might have improved wound healing [36].

Another possible mechanism is that in diabetic wound conditions, cells undergo phagocytosis and produce a large number of proteinases and ROS molecules. Even though ROS molecules contribute to wound healing, in diabetic wound conditions, there will be an uncontrolled production of ROS by auto-oxidation of glucose and glycosylation of scavenging enzymes and depletion of low molecular antioxidant, resulting in oxidative stress [14]. The MDA level is the indicator for ROS generation because it represents the redox reaction level in the cell. In contrast, catalase levels represent the cellular defence, as it is a natural scavenging molecule of H_2_O_2_.

In the present study, we observed elevated MDA levels in the control group and the lower catalase activity. Whereas in the experimental group, we observed optimised catalase and MDA levels. Similar findings were seen in Denadai et al. who treated the diabetic skin wounds of PBMT 6 J/cm^2^ and 660 nm, and Tatmatsu-Rocha et al. who irradiated the super pulsed 904 nm laser of dosage 2.39 J/cm^2^ [37, 38]. It should be noted that these changes were observed in wounded tissues. In contrast, our study estimated the serum levels of catalase and MDA. However, the changes observed were significant.

In diabetic wound conditions, the primary source of ROS is mitochondria. The non-thermal, photochemical reactions of the PBMT optimizes the mitochondrial redox potential of the electron transport chain, which is sensed and transmitted to the cytosol to regulate catalase activity and other enzyme activations. Therefore, PBMT can mediate cell signalling to produce antioxidant molecules to nullify the effect of ROS. Hence, it is evident that the net amount of ROS and antioxidants required for wound healing is facilitated by PBMT, a key for fibroblastic proliferation and angiogenesis in wound healing [39].

In the present study, we observed that, after the irradiation of PBMT of dosage 4 J/cm^2^ to the experimental group, catalase levels were significantly improved, representing a better antioxidant status. In contrast, MDA levels were significantly decreased in post-PBMT to diabetes-induced neuropathic wounds. Therefore, these may serve as adjuvant markers for oxidative status in diabetic wound conditions.

### Strength and Limitations

The present study evaluates the serum levels of oxidative stress markers whose evidence can be translated into clinical research. However, the analysis of these markers in each phase of the wound healing process is not done. The histological findings were taken during the final stages of wound healing. However, it can be considered at inflammatory, proliferative, and remodelling phases for further evidence.

## Conclusion

PBMT of dosage 4 J/cm^2^ enhanced the overall wound healing dynamics in the diabetes-induced neuropathic excisional wound model. PBMT has improved the oxidative status of the wound with optimal changes in catalase and MDA levels and facilitated a faster healing process. We observed an accelerated rate of wound contraction and reduction in the mean healing time required for wound closure compared to the un-irradiated control group. In addition, PBMT also enhanced the skin epithelialization, keratinisation, and proliferation of diabetes-induced neuropathic wounds.

### Future Direction

At present, in clinical practice, the management of diabetic foot ulcer and their associated complications are challenging to clinicians. Based on the present study findings, we recommend that PBMT can be one of the promising adjuvant modality in clinical practice. Based on our findings with markers of oxidative stress, may give a clear direction for the wound condition and its response to the PBMT. However, we recommend conducting further research with different doses and wavelengths.

## Data Availability

The data that support the findings of this study are available from the corresponding author, GAM, upon reasonable request

## Abbreviations

ATP: adenosine triphosphate
DFU: diabetic foot ulcer
DM: diabetes mellitus
DPN: diabetic peripheral neuropathy
LLLT: low-level laser therapy
MDA: malondialdehyde
PBMT: photobiomodulation Therapy
ROS: reactive oxygen species
STZ: streptozotocin
